# A wheat transcription factor positively sets seed vigour by regulating the grain nitrate signal

**DOI:** 10.1111/nph.16234

**Published:** 2019-11-13

**Authors:** Wenjing Li, Xue He, Yi Chen, Yanfu Jing, Chuncai Shen, Junbo Yang, Wan Teng, Xueqiang Zhao, Weijuan Hu, Mengyun Hu, Hui Li, Anthony J. Miller, Yiping Tong

**Affiliations:** ^1^ The State Key Laboratory for Plant Cell and Chromosome Engineering Institute of Genetics and Developmental Biology The Innovative Academy of Seed Design Chinese Academy of Sciences Beijing 100101 China; ^2^ University of Chinese Academy of Sciences Beijing 100049 China; ^3^ CAS‐JIC Centre of Excellence for Plant and Microbial Science (CEPAMS) Shanghai Institutes for Biological Sciences Chinese Academy of Sciences (CAS) Shanghai 200032 China; ^4^ Department of Metabolic Biology John Innes Centre Norwich Research Park Norwich NR4 7UH UK; ^5^ The Institute for Cereal and Oil Crops Hebei Academy of Agriculture and Forestry Sciences Shijiazhuang 050035 China

**Keywords:** NAC transcriptional factor, NRT2, NUE, seed vigour, wheat

## Abstract

- Seed vigour and early establishment are important factors determining the yield of crops. A wheat nitrate‐inducible NAC transcription factor, *TaNAC2*, plays a critical role in promoting crop growth and nitrogen use efficiency (NUE), and now its role in seed vigour is revealed. A TaNAC2 regulated gene was identified that is a NRT2‐type nitrate transporter *TaNRT2.5* with a key role in seed vigour.
- Overexpressing *TaNAC2‐5A* increases grain nitrate concentration and seed vigour by directly binding to the promoter of *TaNRT2.5‐3B* and positively regulating its expression. *TaNRT2.5* is expressed in developing grain, particularly the embryo and husk.
- In *Xenopus* oocyte assays TaNRT2.5 requires a partner protein TaNAR2.1 to give nitrate transport activity, and the transporter locates to the tonoplast in a tobacco leaf transient expression system. Furthermore, in the root *TaNRT2.5* and *TaNRT2.1* function in post‐anthesis acquisition of soil nitrate. Overexpression of *TaNRT2.5‐3B* increases seed vigour, grain nitrate concentration and yield, whereas RNA interference of *TaNRT2.5* has the opposite effects*.*
- The *TaNAC2‐NRT2.5* module has a key role in regulating grain nitrate accumulation and seed vigour. Both genes can potentially be used to improve grain yield and NUE in wheat.

## Introduction

Seed vigour is critical for rapid and uniform crop establishment and, ultimately, yield performance. Improving vigour remains a primary objective of the agricultural industry and breeders (Rajjou *et al*., 2012; Finchsavage & Bassel, 2016). Speed and uniformity of germination are important for defining seed vigour (Finchsavage & Bassel, 2016), and are determined by the growth conditions of the mother plant, genotype and storage conditions (Rajjou *et al.*, 2012; Finchsavage & Bassel, 2016). It has been shown that seeds from the mother plant grown under high nitrogen (N) supply had a faster and higher percentage germination when compared with those grown under low N conditions in *Arabidopsis* (Alboresi *et al.*, 2010; He *et al.*, 2016), wheat (Naylor, 2010) and rice (Hara & Toriyama, 1998). Exogenously supplied nitrate can stimulate seed germination in many plant species (Hendricks & Taylorson, 1974; Alboresi *et al.*, 2010). The identification of seed‐specific nitrate transporters underlines the importance of nitrate provided by the mother plant to the developing seed for subsequent germination and vigour. Overexpression of a seed‐specific high‐affinity nitrate transporter *AtNRT2.7* in *Arabidopsis* increased seed nitrate concentration and germination, whereas knockout of this gene has the opposite effect (Chopin *et al.*, 2007). The low‐affinity nitrate transporter *AtNRT1.6* is involved in delivering nitrate from maternal tissue to the developing embryo in *Arabidopsis*, and a mutation in the gene reduces nitrate accumulation in mature seeds and increases seed abortion rate (Almagro *et al.*, 2008).

The two hormones, abscisic acid (ABA) and gibberellins (GAs), play important roles in controlling seed dormancy and germination, ABA acts as a positive regulator of dormancy induction and maintenance, but as a negative regulator of germination; whereas GA counteracts ABA effects (Kucera *et al.*, 2005). Both exogenous and endogenous nitrate can reduce ABA concentrations during seed imbibition by upregulating the ABA degradation gene *CYP707A2* (Matakiadis *et al.*, 2009; Carrillo‐Barral *et al.*, 2014; Yan *et al.*, 2016), and increasing bioactive GA concentrations by upregulating the biosynthesis of bioactive GA gene *GA3ox2*, to release seed dormancy and stimulate seed germination (Carrillo‐Barral *et al.*, 2014). Mutation in *CYP707A2* impairs nitrate‐promoted germination (Matakiadis *et al.*, 2009; Yan *et al.*, 2016). In a study screening *Arabidopsis* for mutants defective in the response of germination to nitrate, the transcription factor NIN‐like protein 8 (NLP8) was found to be essential for nitrate‐promoted seed germination (Yan *et al.*, 2016). NLP8 positively regulates *CYP707A2* expression, and thus reduces ABA concentrations in seeds. Previous studies found that nitrate could produce nitric oxide (NO), which also stimulates *CYP707A2* expression and seed germination (Bethke *et al.*, 2007; Zheng *et al.*, 2009; Arc *et al.*, 2013). However, NLP8 functions in nitrate‐promoted seed germination even in a nitrate reductase (NR)‐deficient mutant background (Yan *et al.*, 2016). As such, nitrate‐promoted germination is triggered directly by nitrate signaling, but not by nitrate‐assimilation and its products. In fact, it has been documented by many authors that nitrate is a major signal in regulating plant growth, and in breaking seed dormancy independent of its reduction by NR (Hilhorst & Karssen, 1989; Matakiadis *et al.*, 2009; Alboresi *et al.*, 2010; Bewley *et al.*, 2013). However, little is known about the regulation pathway for seed nitrate storage that will later trigger germination in crops.

The present authors found previously that the nitrate‐inducible NAC transcription factor TaNAC2‐5A controls nitrate signaling in wheat. TaNAC2 positively regulates expression of nitrate transporters and root nitrate influx rate and is valuable in breeding wheat with improved yield and N use efficiency (He *et al.*, 2015). It is reported herein that TaNAC2‐5A directly regulates expression of the grain nitrate transporter *TaNRT2.5‐3B*. Overexpression of either *TaNAC2‐5A* or *TaNRT2.5‐3B* increases both grain nitrate concentration and seed vigour, whereas knockdown of *TaNRT2.5* had the opposite effect. Furthermore, overexpression of *TaNRT2.5‐3B* improved grain yield and N accumulation in wheat.

## Materials and Methods

### Plant materials and vector construction

Wheat variety Longchun 23 (LC23) was used as wild‐type (WT) in the present study. *TaNAC2‐5A* overexpression lines, such as TaOE1 and TaOE2 were reported previously (He *et al.*, 2015). To generate *TaNRT2.5‐3B* overexpression lines and *TaNRT2.5* RNAi lines, *TaNRT2.5‐3B* CDS was inserted into the *pUbi‐163* vector, resulting in the construct *pUbi::TaNRT2.5‐3B‐OE;* selected consensus sequence of *TaNRT2.5* was inserted into the *pUbi‐RNAi vector*, resulting in the construct *pUbi::TaNRT2.5‐3B‐RNAi.* The two constructs were transformed into immature embryos of LC23 via the particle bombardment method (Shan *et al.*, 2014). For overexpression, OE102‐6 and OE103‐1 are two independent *TaNRT2.5‐3B* overexpression lines from five positive selected lines. For RNAi, R100‐1 and R109‐2 are two independent RNAi lines from four positive selected lines. The primers used for vector construction are listed in Supporting Information Table S1.

### Wheat growth conditions

The WT, *TaNAC2‐5A* overexpression lines and *TaNRT2.5* transgenic lines were used in the hydroponic culture, soil pot and field experiments. The nutrient solution composition, methods for seed sterilization and germination and growth conditions for the hydroponic experiments were described previously (He *et al.*, 2015). The pot seed germination experiments used a calcareous soil that was collected from the experimental station of the Institute of Genetics and Developmental Biology in Beijing. The soil was wetted to 20% soil water content, and 100 seeds of the WT and the transgenic lines were sowed in each pot at 20°C. Photographs were taken and the shoot length of seedlings were measured at 7 d after sowing.

The field experiment was conducted at the experimental station of the Institute for Cereal and Oil Crops, Hebei Academy of Agriculture and Forestry Sciences, Hebei Province, China. The experiment had a random block design with four replications. The fertilizer supply was 18 g m^−2^ nitrogen (N) in the form of urea, with 12 g m^−2^ applied before sowing and 6 g m^−2^ applied at the stem elongation stage, and 13.5 g m^−2^ phosphorus (calcium superphosphate) applied before sowing. Total biomass yield per plant, grain yield per plant, spike number per plant, and grain number of the primary spike were recorded for 30 representative plants for each sample group. The 1000‐grain weight was determined according to the DW of 500 dried grains.

### Seed germination assays on Petri dishes

Seeds collected at the same time were used for germination assays. The germination assays were performed at least one month after the seeds were harvested. Four independent biological replicates were used, and each replicate included 100 seeds. Seeds were surface‐sterilized with 30% H_2_O_2_ for 10 min and washed five times with sterile water, then were sown on 10 × 10 cm Petri dish with filter paper containing 10 ml saturated CaSO_4_ solution at 20°C. Germination was scored at the indicated time points and scored as positive when the radicle protruded from the seed.

### Controlled deterioration test (CDT) and tetrazolium assay

The CDT was performed with some modifications (see Zuo *et al.*, 2018). Briefly, a closed container was used with saturated KCl solution to reach 82% relative humidity. Seeds were equilibrated for 4 d at 25°C in the dark. Thereafter the seeds were artificially aged for 2 d at 82% relative humidity and at 45°C in the dark. Seeds were dried at room temperature for 1 d before the tetrazolium assay.

Tetrazolium assay was performed in three biological repeats as described by Salvi *et al.* (2016). Wheat seeds were initially sterilized with sodium hypochlorite solution (v/v) and then washed with sterilized distilled water for five times. Sterilized seeds were then incubated in 1% tetrazolium solution in darkness at 30°C for 48 h to stain. The staining patterns were observed under the anatomical microscope. Wheat seed with embryos totally or mostly stained to red were defined as viable seeds. Seed vigour = viable seed number/total seed number × 100%.

### Nitrate concentration measurements

Grain nitrate concentrations were determined as described previously (Chopin *et al.*, 2007). The whole wheat seeds or razor‐dissected grains were homogenized into powder and then 1 ml 80% (w/v) ethanol was added into 250 mg wheat seed powder at 4°C and extracted for 2 h. Nitrate content of seeds was analyzed by high‐performance liquid chromatography on an ICS‐5000 analyzer (Thermo Fisher, Waltham, MA, USA).

The nitrate concentrations in leaves or roots of wheat seedlings were measured by the salicylic acid (SA) concentrated sulfuric acid colorimetry method. Samples of 1 g fresh wheat leaves or roots were homogenized, and 10 ml sterile water was added, and then nitrate was extracted for 30 min in a boiling water bath. 0.1 ml extracted solution, 0.4 ml 5% SA‐concentrated sulfuric acid (w/v) solution and 9.5 ml 8% NaOH (w/v) solution were well mixed and OD_410_ was measured when the solution was cooled to 25°C. the nitrate concentration of the extracted solution was calculated using a standard curve.

### Quantitative real‐time (qRT‐)PCR

Total RNA from plant tissues was extracted with the Plant RNeasy Kit (Qiagen). First‐strand complementary DNA was synthesized from 2 μg DNase I‐treated total RNA using the PrimeScript RT Reagent Kit (TaKaRa, Basel, Switzerland) according to the manufacturer’s instructions. The qRT‐PCR analysis was performed with a LightCycler 480 engine (Roche) using Light‐Cycler480 SYBR Green I Master Mix (Roche). The primers used for qRT‐PCR are detailed in Table S2.

### Chromatin immunoprecipitation (ChIP) assay

The LC23 wheat seedlings were used for ChIP assays performed according to methods described previously (Bowler *et al.*, 2004). Anti‐TaNAC2 antibody was used to precipitate the DNA–TaNAC2 complexes. The DNA was released with proteinase K and then purified for further PCR analysis. The enrichment of DNA fragments was determined using qRT‐PCR, with the primers detailed in Table S2.

### Fusion protein preparation and electrophoretic mobility shift assays (EMSAs)

The full‐length CDS of *TaNAC2‐5A* were cloned into the *pGEX‐6p‐1* vector. The recombinant GST‐fusion proteins were expressed in *Escherichia coli* BL21 (*Transseta*) and purified to homogeneity using GE sepharose 4B beads (GE, Boston, MA, USA). Oligonucleotide probes were synthesized and labeled with biotin at their 5’ ends (Invitrogen). EMSAs were performed using the LightShift Chemiluminescent EMSA Kit (Thermo Fisher Scientific, Waltham, MA, USA). The primers used for vector construction are listed in Table S1.

### Assay of nitrate transporter activity in *Xenopus laevis* oocytes

The CDSs of *TaNRT2.5‐3A*/*B*/*D*, *TaNAR2.1‐6B*, *TaNAR2.2‐5B* and *TaNAR2.3‐6B* were amplified and cloned into the oocyte expression vector *pT7Ts* between the restriction sites *Spe*I and *Bgl*II and then linearized with *Eco*RI. Capped mRNA was synthesized *in vitro* using the mMESSAGE mMACHINE kit (Ambion, AM1340) according to the manufacturer’s protocol. *Xenopus laevis* oocytes at stage V–VI were injected with 50 ng cRNA per oocyte. After injection, oocytes were cultured in MBS medium for 48 h and then used for ^15^N‐NaNO_3_‐uptake assays; 500 µM ^15^N–NaNO_3_ was used for the uptake assays, as described previously (Tong *et al.*, 2005). The primers used for vector construction are listed in Table S1.

### Subcellular localization and Western blot

The CDSs of *TaNRT2.5‐3B* and *TaNAR2.1‐6B* were fused in frame with green and red fluorescent proteins (GFP and RFP) via cloning into the binary vector pMDC83‐CaMV35S‐GFP and *pJAH2044‐CaMV35S‐RFP*. The resulting vectors were transformed into *Agrobacterium* strain GV3101 as GV3101‐TaNRT2.5‐3B and GV3101‐TaNAR2.1‐6B. Then GV3101‐TaNRT2.5‐3B and GV3101‐TaNAR2.1‐6B were co‐infiltrated or separately infiltrated into tobacco leaf epidermal cell. The GFP and RFP image was observed with a confocal microscope (Zeiss LSM 710 NLO). The primers used for vector construction are listed in Table S1. Eight gram infiltrated tobacco leaves of different combined GV3101 were used for Western blot assays. The membrane protein fractionation and Western blot assay methods were as described previously (Ueno *et al.*, 2010).

### ^15^N‐nitrate influx, uptake rate and accumulation after anthesis assay

^15^N‐nitrate influx and uptake rate were determined using a ^15^N‐KNO_3_ assay. The ^15^N content of wheat seedling root and shoot was determined after 15 min or 12 h of uptake of ^15^N‐KNO_3_. ^15^N‐nitrate influx and uptake rate were calculated as the amount of ^15^N taken up per unit weight of roots per unit time. ^15^N‐nitrate influx and uptake rate assays were performed using 200 µM and 2 mM ^15^N‐KNO_3_, respectively. Roots and shoots were collected and dried at 70°C. Samples were ground and the ^15^N content was determined using an isotope ratio mass spectrometer with an elemental analyzer (Isoprime 100‐EA, Manchester, UK). To determine the ^15^N‐nitrate accumulation after anthesis, the field experiment was fertilized with ^15^N‐KNO_3_. The ^15^N‐nitrate accumulation was calculated as the amount of ^15^N taken up per plant in grain and straw. The N accumulation after anthesis from fertilizer also was determined as the ratio of the amounts of ^15^N to total N in grain and whole plant, and the ^15^N content was determined as described above.

### Statistical analysis of data

Statistically significant differences using Spss 17.0 for Windows (Spss, Armonk, NY, USA) were computed based on Student’s *t*‐tests.

## Results

### Overexpressing *TaNAC2‐5A* increases grain nitrate concentration and seed vigour

**Figure 1:**
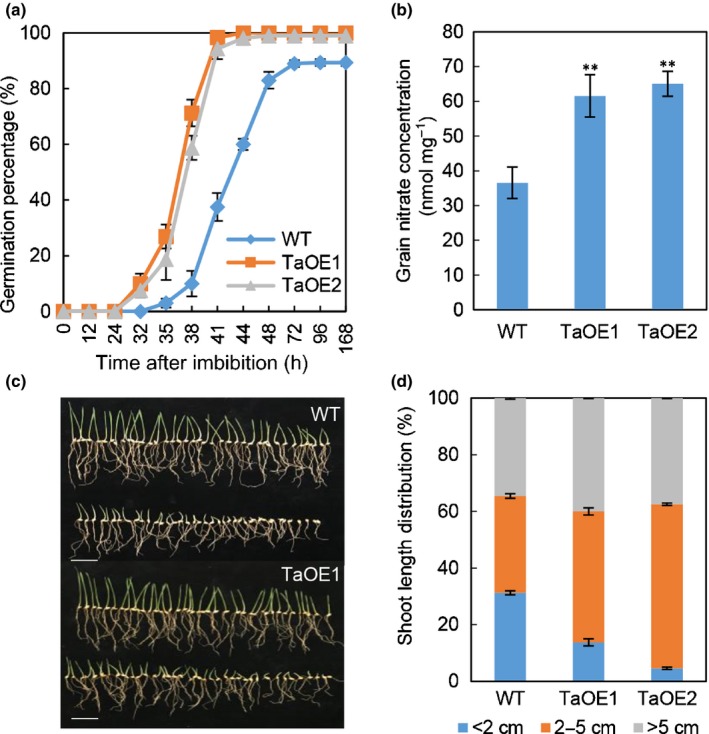
Overexpression of *TaNAC2‐5A* increases seed vigour and grain nitrate concentration. (a) Germination percentage of *TaNAC2‐5A* overexpression lines and wild‐type (WT) in Petri dishes. Values are means ± SE (*n* = 4). **, *P* < 0.01 (Student’s *t*‐test). (b) Grain nitrate concentration measured using the dry seeds. Values are means ± SE (*n* = 4). **, *P* < 0.01 (Student’s *t*‐test). (c) Seedling images of germination assay in pot experiment. Bars, 5 cm. (d) Shoot length (cm) distribution of (c). Values are means ± SE (*n* = 4).

The present authors’ previous study showed that the NAC transcription factor *TaNAC2‐5A* is nitrate inducible and overexpression of *TaNAC2‐5A* increases root nitrate influx rate, N uptake and grain yield in wheat (He *et al.*, 2015). It was observed herein that overexpression of *TaNAC2‐5A* increased the speed and rate of germination in Petri dishes and also the seed vigour in the CDT experiment (Figs 1a, S1a). When the seeds were germinated in soil, the overexpression lines had significantly fewer seedlings with < 2 cm shoots when compared with WT (Figs 1c, d, S1b), indicating the transgenic lines grew more rapidly and uniformly established seedlings. As seed nitrate is a signal promoting germination and *TaNAC2‐5A* is nitrate‐responsive, the nitrate concentrations were measured and compared in the grains of the *TaNAC2‐5A* overexpression lines and WT. Grain nitrate concentration was increased by *c*. 71% in *TaNAC2‐5A* overexpression lines compared with WT (Fig. 1b), but overexpression only slightly increased total N concentration (He *et al.*, 2015).

*Arabidopsis* seeds from plants grown under high N supply had higher nitrate concentrations and germination rates than those from plants grown under low N conditions (Chopin *et al.*, 2007; Rajjou *et al.*, 2012). This also was observed in the wheat variety LC23 (WT for the transgenic lines) as the seeds harvested from high N treatment had a three‐fold higher nitrate concentration and 20% higher germination than those from low N treatment (Fig. S2a, b). Seed germination was assayed in the mini‐core collections of Chinese wheat varieties (Wang *et al.*, 2012) and the three fastest germinating varieties were found to have much higher grain nitrate concentrations (Fig. S2c, d), and higher *TaNAC2* expression in the germinating seeds when compared with the three slower ones (Fig. S2e).

Taken together, these results show that wheat grain nitrate concentration impacts on germination and overexpression of *TaNAC2‐5A* increased grain nitrate concentration resulting in rapid and uniform germination.

### Identifying the downstream genes of TaNAC2

**Figure 2:**
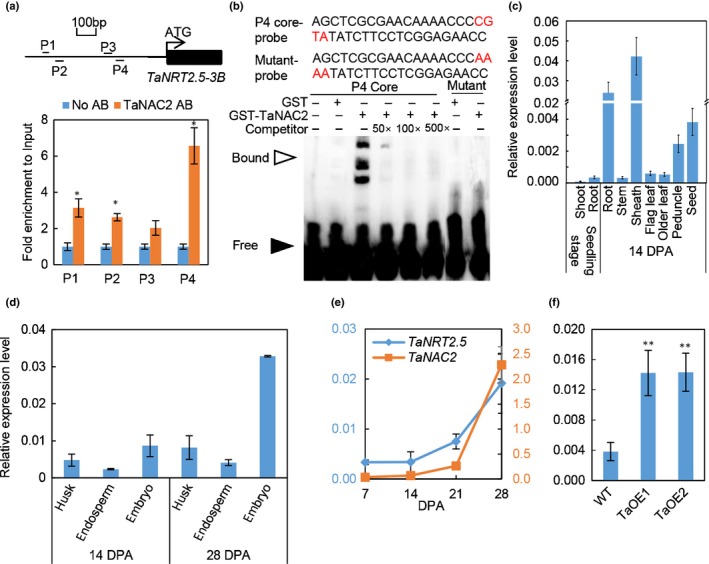
TaNAC2‐5A binds to the promoter of *TaNRT2.5‐3B* and regulates the expression. (a) Chromatin immunoprecipitation quantitative PCR (ChIP–qPCR) assay of TaNAC2‐5A binding to *TaNRT2.5‐3B* promoter. P1–P4 are fragments selected in *TaNRT2.5‐3B* promoter for ChIP–qPCR analysis. (b) Electrophoretic mobility shift assay (EMSA) of TaNAC2‐5A binding to P4 fragment from (a). (c) The expression pattern of *TaNRT2.5* in different organs of wheat at seedling stage and 14 d post‐anthesis (DPA). (d) The expression pattern of *TaNRT2.5* in different parts of wheat seeds in 14 and 28 DPA. (e) The expression pattern of *TaNAC2* and *TaNRT2.5* in developing seeds at 7, 14, 21 and 28 DPA. (f) The expression level of *TaNRT2.5* in seeds of *TaNAC2‐5A* overexpression lines and wild‐type (WT). Significance for the difference between the means of the transgenic lines and WT: **, *P* < 0.01 (Student’s *t*‐test). *TaActin* was used as an internal reference. Data represented as means ± SE (*n* = 4).

It is reported that *TaNRT2.1‐6B*, *TaNPF7.1‐6D* and *TaGS2‐2A* are directly and positively regulated by *TaNAC2* (He *et al.*, 2015), but these genes were not co‐expressed with *TaNAC2* in the developing seeds during the grain‐filling stage herein (Fig. S3a). Other unknown downstream genes of *TaNAC2* may be involved in delivering nitrate to the grain. To understand the mechanism of *TaNAC2* control of grain nitrate concentration and germination, Chromatin Immunoprecipitation Sequencing (ChIP‐Seq) was performed using whole plants 2 d after germination. In total, ChIP‐Seq identified 61 candidate genes, including *TaNRT2.5‐3B* (Table S3). Further ChIP‐qPCR analysis showed that TaNAC2 could bind to the promoter of *TaNRT2.5‐3B* (Fig. 2a), *TaLAX1‐3B* and *‐1D*, and *TaARR12‐6A* (Fig. S4). *TaLAX1‐3B* and *‐1D* were predicted to encode auxin influx transporters, and *TaARR12‐6B* is a homologue of *AtARR10*/*12* involving the cytokinin signaling. Among these newly identified downstream genes of *TaNAC2‐5A*, *TaNRT2.5* was mainly expressed in the maturing seed (Fig. S3b–h) and was predicted to encode a putative high‐affinity NRT2‐type nitrate transporter. A more detailed analysis was then performed to determine whether *TaNAC2* increased grain nitrate concentration and germination by regulating *TaNRT2.5*.

### *TaNRT2.5* works downstream of TaNAC2

Phylogenetic analysis of NRT2s from wheat, rice, maize, barley, soybean and *Arabidopsis* showed that the NRT2 proteins appeared to be clustered into two main monophyletic groups, a larger Group A and a smaller Group B which contained TaNRT2.5s (Fig. S5).

A DNA EMSA was conducted to confirm the result in ChIP‐qPCR (Fig. 2a). As shown in Fig. 2(b), the GST‐TaNAC2 fusion proteins could bind biotin‐labeled DNA probes in *TaNRT2.5‐3B* promoter (*proTaNRT2.5‐3B*). Moreover, the addition of an excess amount of unlabeled *proTaNRT2.5‐3B* probe effectively reduced the binding of GST‐TaNAC2 to labeled DNA probe. The parallel experiment indicated that GST‐TaNAC2 was unable to bind a mutant form of the biotin‐labeled *proTaNRT2.5‐3B* probe (Fig. 2b). These results indicated that TaNAC2‐5A could bind to *proTaNRT2.5‐3B in vivo* and *in vitro*.

Next the tissue‐specific transcript of *TaNRT2.5* was checked, revealing that there was higher expression in roots, leaf sheath, peduncle and developing seeds in wheat plants at 14 d post‐anthesis (DPA) (Fig. 2c). Measurements of the transcript level of *TaNRT2.5* in dissected grains at 14 and 28 DPA showed that *TaNRT2.5* was expressed mainly in the husk and embryo (Fig. 2d), which is similar to the nitrate distribution (Fig. S6a). The expression of both *TaNAC2* and *TaNRT2.5* in seeds also was found to be increased during grain filling (Fig. 2e), and overexpression of *TaNAC2‐5A* greatly upregulated *TaNRT2.5* expression in seeds compared to WT (Fig. 2f). A hydroponic culture of seedlings was used to show that the root expression of *TaNRT2.5* was nitrate inducible, and upregulated by low N availability (Fig. S7a, b), as has been shown for *TaNAC2* (He *et al.*, 2015)*.* However, the response of *TaNAC2* to nitrate induction was earlier than that of *TaNRT2.5* (Fig. S7a). Taken together, *TaNAC2* and *TaNRT2.5* are co‐expressed in developing seeds, and *TaNAC2* directly and positively regulates *TaNRT2.5* expression. No significant differences in the expression patterns of *TaNAC2* and *TaNRT2.5* homeologs could be detected in the gene atlas (see Fig. S8).

### TaNRT2.5‐TaNAR2.1 has nitrate transport activity and locates mainly on tonoplast

Sequence analysis predicts TaNRT2.5‐3B to be a high‐affinity nitrate transporter. As some NRT2 members require a partner protein NAR2, for nitrate transport at relatively low concentration ranges (Tong *et al.*, 2005; Orsel *et al.*, 2006; Feng *et al.*, 2011), a search was carried out for TaNAR2s from the wheat genome database (http://plantSEnsembl.org/Triticum_aestivum/Info/Index) which were named according to their relationship to HvNAR2s in barley (Tong *et al.*, 2005). Phylogenetic analysis showed that the NAR2 proteins clustered into three clades which included TaNAR2.1s, TaNAR2.2s and TaNAR2.3s, respectively (Fig. S7c).

**Figure 3:**
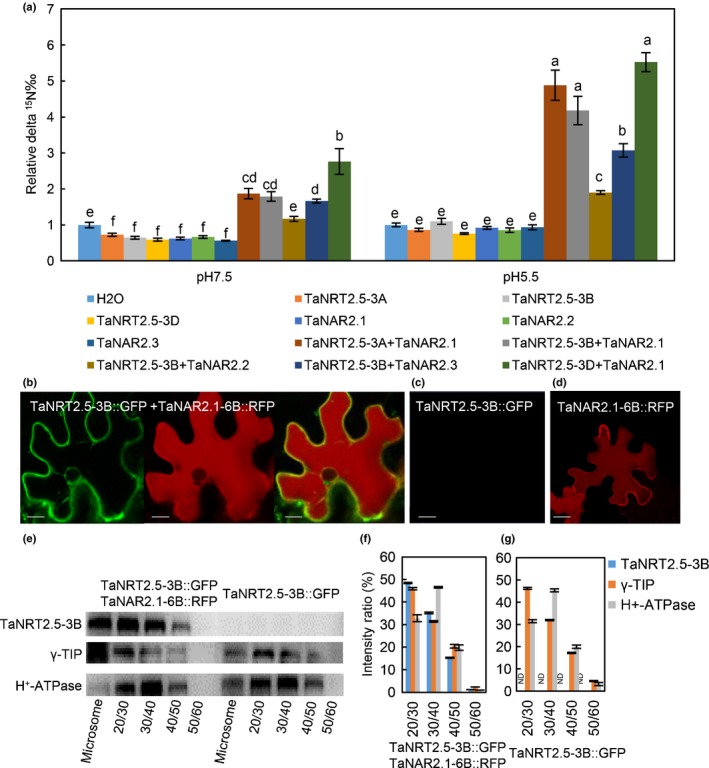
The nitrate transport activity and subcellular localization of TaNRT2.5. (a) The nitrate transport activity of different combinations of TaNRT2.5s and TaNAR2s in pH 7.5 and 5.5 relative to water‐injected controls. Uptake from 500 µM of ^15^N labelled nitrate in 6 h was used for *Xenopus* oocytes which were injected with water or different cRNA combinations. The delta‐^15^N values are shown as the mean ± SE for four oocytes. Different letters above the columns indicate statistically significant differences at the *P* < 0.05 level according to one‐way ANOVA. (b–d) Fluorescence of green fluorescent protein (GFP) in tobacco leaf epidermal cells expressing *TaNRT2.5‐3B::GFP* coupled with *TaNAR2.1‐6B::RFP* (b), *TaNRT2.5‐3B::GFP* alone (c) or *TaNAR2.1‐6B::RFP* (d) (Bars, 20 μm)*.* (b) Left, TaNRT2.5‐3B::GFP signal; middle, TaNAR2.1‐6B::RFP signal; right, overlap of these two signals. (c) TaNRT2.5‐3B::GFP signal. (d) TaNAR2.1‐6B::RFP signal. (e–g) Microsome from whole leaf of expressed TaNRT2.5‐3B::GFP coupled with TaNAR2.1‐6B::RFP or TaNRT2.5‐3B::GFP alone were extracted and fractionated by sucrose‐density gradient centrifugation, then the different fractions were used for Western blot. Antibodies of anti‐GFP, anti‐γ‐TIP (tonoplast marker), and anti‐H^+^‐ATPase (plasma membrane marker) were used (e). Relative band intensity was measured using imagej software (NIH, Bethesda, MD, USA). The ratio of different band intensity to total intensity was calculated, and three independent experiments were performed. Data represented as means ± SE (*n* = 3) (f, g).

The nitrate transport activity of TaNRT2.5 expressed in *Xenopus laevis* oocytes was tested by injecting oocytes with nuclease‐free water, *TaNRT2.5‐3B* cRNA alone and *TaNRT2.5‐3B* cRNA together with three different *TaNAR2s* cRNAs. Figure 3(a) shows that only oocytes co‐injected with *TaNRT2.5‐3B* and *TaNAR2.1‐6B* in combination accumulated significantly more ^15^N‐nitrate than the control oocytes. Further analysis revealed that all the homeolog genes of TaNRT2.5s (*TaNRT2.5‐3A*, *‐3B* and *‐3D*) required *TaNAR2.1‐6B* for nitrate transport activity. The NRT2 transport mechanism is generally thought to be cotransport with protons and significantly higher nitrate transport activity was measured in oocytes injected with *TaNRT2.5‐3B/TaNAR2.1‐6B* cRNAs incubated at pH 5.5 when compared with pH 7.5 (Fig. 3a). The predicted protein sequences of the three genes were very similar (Fig. S7e). Interestingly, among the TaNAR2s only the transcript of *TaNAR2.1* was detected in seeds and mainly in the embryo (Fig. S7d).

In order to investigate the subcellular localization of TaNRT2.5‐3B, the tobacco (*Nicotiana benthamiana*) leaf transient expression system was used. When *TaNRT2.5‐3B::GFP* and *TaNAR2.1‐6B::mRFP* were co‐infiltrated, a strong GFP signal was detected which seemed to localize to the tonoplast and on membranes around the nucleus (Fig. 3b). When *TaNRT2.5‐3B::GFP* was expressed alone, the GFP signal could not be detected under the same microscopy conditions (Fig. 3c). To confirm the tonoplast location of the TaNRT2.5‐3B and TaNAR2.1‐6B complex, membrane proteins were extracted and separated into different fractions using sucrose‐density gradient centrifugation. Western blot analysis showed that TaNRT2.5‐3B::GFP was present mainly in the same fraction as the anti‐γ‐TIP (a tonoplast marker), but in different fractions from the anti‐H^+^‐ATPase (a plasma membrane marker), when the tobacco leaves were co‐infiltrated with *TaNRT2.5‐3B::GFP* and *TaNAR2.1‐6B::mRFP* (Fig. 3e, f). By contrast, when *TaNRT2.5‐3B‐GFP* was expressed alone, the TaNRT2.5‐3B::GFP was not detected in any of the fractions (Fig. 3e, g). Taken together, these data suggest that TaNRT2.5‐3B was localized to the tonoplast when co‐expressed with TaNAR2.1‐6B, but TaNRT2.5‐3B alone seemed unstable.

### *TaNRT2.5* positively affects seed vigour and grain nitrate concentration

**Figure 4:**
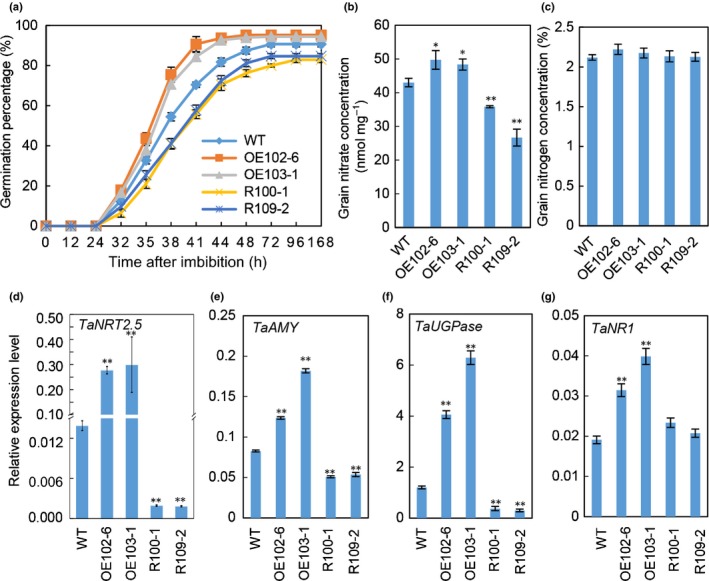
Overexpression of *TaNRT2.5‐3B* increases seed germination, grain nitrate concentration and modifies the expression of some associated genes. (a) Germination percentage of *TaNRT2.5* transgenic lines and wild‐type (WT). (b) Grain nitrate concentration of *TaNRT2.5* transgenic lines and WT. (c) Grain nitrogen concentration of *TaNRT2.5* transgenic lines and WT. (d–g) Expression levels of *TaNRT2.5* (d), *TaAMY* (e), *TaUGPase* (f) and *TaNR1* (g) in the germinating seeds (60 h after sowing). OE102‐6 and OE103‐1, overexpression lines; R100‐1 and R109‐2, RNAi lines. *TaActin* was used as an internal reference. Data represented as means ± SE (*n* = 4). Significance for the difference between the means of the transgenic lines and WT: *, *P* < 0.05; **, *P* < 0.01 (Student’s *t*‐test).

Because *TaNAC2‐5A* affected seed vigour and directly regulated *TaNRT2.5*, it was tested whether *TaNRT2.5* could affect germination and grain nitrate concentration. Transcript analysis of *TaNRT2.5* confirmed an increase in the *TaNRT2.5‐3B* overexpression lines and knock down in the *TaNRT2.5* RNAi lines (Fig. 4d). Petri dish germination assays and the CDT both showed that the *TaNRT2.5‐3B* overexpression lines germinated earlier and finally achieved a higher germination percentage than WT seeds, whereas *TaNRT2.5* RNAi lines had the opposite phenotype (Figs 4a, S9a, b). Overexpressing *TaNRT2.5‐3B* also increased germination, whereas knock‐down of *TaNRT2.5* reduced seed vigour when germinated in soil (Fig. S9c–h). The nitrate concentration was measured in the seed used in the germination assays, revealing that the *TaNRT2.5‐3B* overexpression lines had significantly higher, and the RNAi lines lower grain nitrate concentrations compared with WT (Fig. 4b). The nitrate concentration was increased in the dissected parts of *TaNRT2.5* transgenic lines by overexpression, but was decreased in the husk and embyro of the *TaNRT2.5* RNAi lines (Fig. S6b), probably caused by the promoter expression patterns of *TaNRT2.5*. However, there was no significant difference in total grain N concentration between WT and the transgenic lines (Fig. 4c).

In order to better understand how altering *TaNRT2.5* expression affected germination, the expression of *TaAMY* encoding a starch‐degrading α‐amylase, *TaUPGase* encoding a UDP‐glucose pyrophosphorylase, and *TaNR1* encoding a nitrate reductase in germinating seeds were measured 60 h after sowing. When compared with WT, overexpressing *TaNRT2.5‐3B* greatly increased expression of these three genes; however, knockdown of *TaNRT2.5* dramatically reduced expression of *TaAMY* and *TaUPGase*, but did not significantly alter *TaNR1* expression (Fig. 4d–g).

### Overexpressing *TaNRT2.5‐3B* promotes seedling growth and N uptake under hydroponic conditions

**Figure 5:**
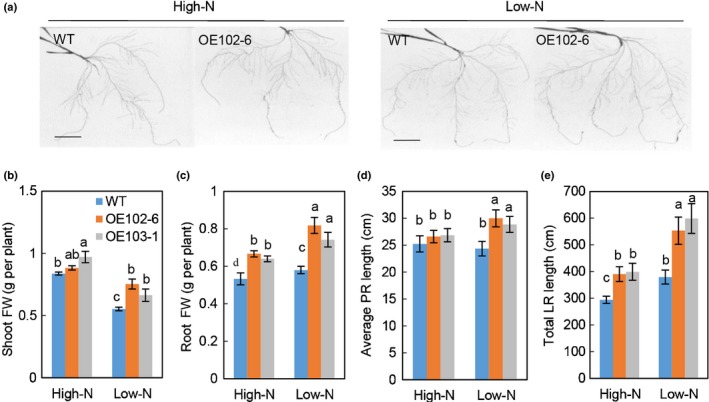
Overexpression of *TaNRT2.5‐3B* promotes wheat seedling growth. (a) Root images of *TaNRT2.5‐3B* overexpression lines (OE102‐6 and OE103‐1) and wild‐type (WT) grown under high and low nitrogen (N) supply conditions using a hydroponic culture system. Bars, 5 cm. (b) Shoot FW; (c) Root FW, (d) Average primary root (PR) length; (e) Total lateral root (LR) length. Data represented as means ± SE (*n* = 4). Different letters above the columns indicate statistically significant differences at the *P* < 0.05 level according to one‐way ANOVA.

As *TaNRT2.5* mRNA was expressed at very low levels in wheat seedlings (Fig. 2c), only overexpression lines were used to investigate the effects of *TaNRT2.5‐3B* on N uptake and seedling growth (see Fig. 5a). Compared with WT, the overexpression lines had significantly higher root and shoot FW under both high and low N supply (Fig. 5b, c). The overexpression lines also displayed longer average primary root (PR) length under low N conditions (Fig. 5d), longer total lateral root (LR) length (Fig. 5e) under both high and low N conditions.

**Figure 6:**
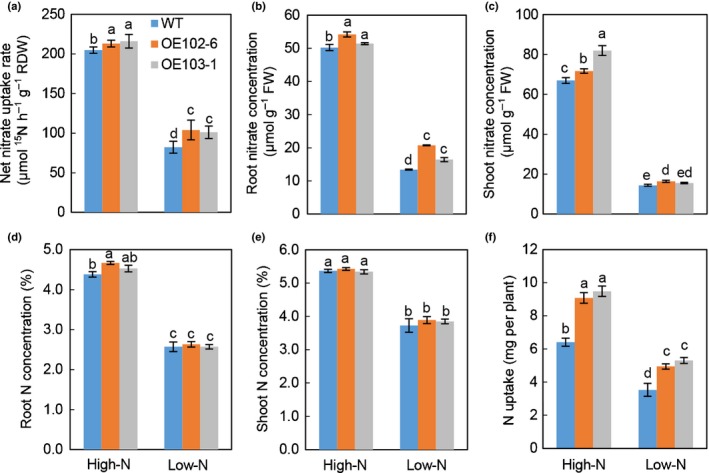
Overexpression of *TaNRT2.5‐3B* promotes nitrate uptake and accumulation at the seedling stage. The seedlings of *TaNRT2.5‐3B* overexpression lines (OE102‐6 and OE103‐1) and wild‐type (WT) grown under high and low nitrogen (N) supply conditions using a hydroponic culture system. (a) Net nitrate uptake rate. (b) Root nitrate concentration. (c) Shoot nitrate concentration. (d) Root N concentration. (e) Shoot N concentration. (f) N uptake. Data represented as means ± SE (*n* = 4). Different letters above the columns indicate statistically significant differences at the *P* < 0.05 level by one‐way ANOVA.

Next the effects of overexpressing *TaNRT2.5‐3B* on nitrate influx were investigated, revelaing no difference in nitrate influx rate between *TaNRT2.5‐3B* overexpression lines and WT when nitrate influx rates were measured with ^15^N‐nitrate at 2 and 0.2 mM for 5 min (Fig. S10). But after the plants were exposed to ^15^N‐nitrate for 12 h, higher net nitrate uptake rates were detected in the *TaNRT2.5‐3B* overexpression lines relative to WT under both high and low nitrate supply (Fig. 6). These increases were more obvious under low nitrate supply (see Fig. 6a). Measurements of plant tissue nitrate revealed that the *TaNRT2.5‐3B* overexpression lines had significantly higher root nitrate concentrations under low nitrate supply and in shoots under high N supply (Fig. 6b, c). The overexpression lines had greater total N uptake under both high and low N conditions (Fig. 6f), but root and shoot N concentrations were not very different (Fig. 6d, e).

### Overexpressing *TaNRT2.5‐3B* increases N uptake and yield under field conditions

A field experiment was carried out to investigate the effects of manipulating *TaNRT2.5* expression on N use and yield performance. The data in Table 1 showed that *TaNRT2.5‐3B* overexpression lines had 16.9–17.9% (*P* < 0.05) higher biomass yield per plant, 19.4–21.3% (*P* < 0.05) higher grain yield per plant, 13.5–15.9% (*P* < 0.05) higher spike number per plant, 17.8–21.3% (*P* < 0.05) higher aerial N accumulation than WT, whereas the RNAi lines had the opposite phenotype. There were no significant differences in grain number per spike, 1000‐grain weight, grain and straw N concentrations between the transgenic lines and WT. These results indicated that manipulating *TaNRT2.5* expression altered grain yield mainly by altering spike number and altered aerial N accumulation mainly by altering wheat growth, but not by changing N concentrations in the aerial parts.

**Table 1:** Agronomic traits and nitrogen (N) accumulation of the *TaNRT2.5* overexpression and RNAi lines and wild‐type (WT) in the field experiment.

Trait	WT	OE102‐6	OE103‐1	R100‐1	R109‐2
Biomass (g per plant)	36.58 ± 3.32	42.78 ± 5.49*	43.11 ± 4.49*	32.34 ± 3.75*	32.65 ± 2.56*
Grain yield (g per plant)	12.98 ± 1.66	15.75 ± 1.24*	15.50 ± 1.02*	10.94 ± 1.58*	10.61 ± 1.71*
Spike number	11.96 ± 1.58	13.58 ± 1.88*	13.86 ± 2.17*	9.90 ± 1.56*	10.10 ± 2.46*
Grain number per main spike	65.17 ± 1.41	64.58 ± 1.88	67.22 ± 1.92	58.50 ± 1.88*	62.43 ± 1.41
1000‐grain weight (g)	41.83 ± 0.38	42.95 ± 1.76	41.33 ± 0.89	41.04 ± 0.91	41.16 ± 0.66
Grain N concentration (%)	2.12 ± 0.04	2.22 ± 0.07	2.18 ± 0.06	2.13 ± 0.07	2.13 ± 0.06
Straw N concentration (%)	0.59 ± 0.09	0.60 ± 0.04	0.59 ± 0.03	0.58 ± 0.03	0.59 ± 0.03
Aerial N accumulation (mg per plant)	406.5 ± 41.0	492.9 ± 34.2*	478.7 ± 33.5*	347.3 ± 24.0*	346.2 ± 18.2*

OE102‐6 and OE103‐1, overexpression lines; R100‐1 and R109‐2, RNAi lines. Data were means ± SE of four biological replicates. Each replicate contained ≥ 10 plants. Asterisks indicate statistically significant difference between WT and transgenic line: *,* P* < 0.05 (Student’s *t‐*test).

**Figure 7:**
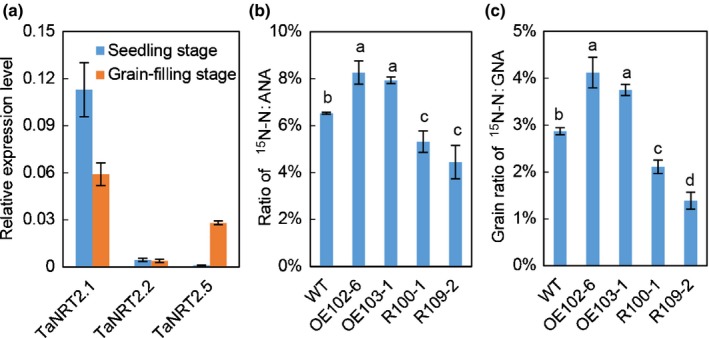
Overexpression of *TaNRT2.5‐3B* increases the contribution of post‐anthesis N uptake to N accumulated in aerial parts and grains. (a) The expression levels of different TaNRT2 family genes in roots of wild‐type (WT) seedling and the plants 14 d post‐anthesis (DPA). *TaActin* was used as an internal reference. (b, c) Ratio of ^15^N‐nitrogen (N) accumulation to ANA (b) and to GNA (c) of the *TaNRT2.5* overexpression (OE102‐6 and OE103‐1) and RNAi (R100‐1 and R109‐2) lines and WT. ANA, aerial N accumulation; GNA, grain N accumulation. Data represented as means ± SE (*n* = 4). Different letters above the columns indicate statistically significant differences at the *P* < 0.05 level according to one‐way ANOVA.

It was observed that the expression of *TaNRT2.5* in roots was much higher at the grain filling stage when compared with the seedling stage (Figs 2c, 7a), and *TaNRT2.5* and the root‐specific *TaNRT2.1* had comparable mRNA abundance in roots at the grain filling stage. Hence, it was questioned whether manipulating *TaNRT2.5* expression affects N uptake and distribution after anthesis. After analyzing the ^15^N–N and total N accumulated in straw and grain of the physiologically matured plants which were treated with ^15^N‐nitrate in the soil at anthesis, the *TaNRT2.5‐3B* overexpression lines were detected to have significantly higher ratios of ^15^N–N over the total N accumulated in aerial parts relative to WT (Fig. 7b). Likewise, the ^15^N–N over total N accumulated in grains (Fig. 7c) compared with WT was significantly higher in overexpression plants, whereas the RNAi lines showed the opposite phenotype. These results suggest that *TaNRT2.5* may be involved in late root N uptake and allocation to the grain after anthesis.

## Discussion

Good germination and subsequent rapid seedling establishment are key features for the successful propagation of plants. We showed that *TaNAC2‐5A* regulated grain nitrate concentration and germination. Furthermore, *TaNAC2* mRNA levels showed a positive correlation (*r* ^2^ = 0.71) with grain nitrate concentration and germination in diverse wheat varieties (Fig. S2). In addition, overexpressing *TaNAC2‐5A* promotes seed germination, and increases grain nitrate concentration (Fig. 1). During germination, seedlings are particularly sensitive and may easily suffer during adverse environmental conditions. Overexpressing *TaNAC2* in *Arabidopsis* has been shown to improve tolerance to drought and salt stress (Mao *et al.*, 2012; Huang & Wang, 2016) and so enhancing *TaNAC2* expression level by traditional breeding or transgenic methods may help wheat to resist these stresses and increase germination rate and improve yield. Nitrate may regulate seed germination by regulating the bioactive abscisic acid (ABA) and gibberellin (GA) content (Kucera *et al.*, 2005; Matakiadis *et al.*, 2009; Carrillo‐Barral *et al.*, 2014). *TaNAC2* is inducible by nitrate, as well as by ABA (Mao *et al.*, 2012; Zhang *et al.*, 2018) and the protein can bind to the promoters of downstream genes which were involved in nitrate transport and assimilation (He *et al.*, 2015), phytohormone transport and some other transcription factors (TFs) (Fig. S4). *TaNAC2* may be involved in the cross‐talk between nitrate and phytohormones in the processes of germination and early seedling establishment.

Multiple lines of molecular and biochemical evidence show that TaNAC2‐5A can bind the promoter of nitrate transporter *TaNRT2.5‐3B* and promote expression of *TaNRT2.5* (Fig. 2). The biochemical data were nicely in agreement with genetic findings. Overexpressing *TaNRT2.5‐3B* promoted germination and increased grain nitrate concentration, but not the total seed nitrogen (N) (Fig. 4), and enhanced spikes and grain yield (Table 1). The NRT2 family size is different in each plant species (Miller *et al.*, 2007; Pellizzaro *et al.*, 2015; Bajgain *et al.*, 2018; Fig. S5), and some NRT2s are capable of nitrate uptake only when co‐expressed with a partner NAR2 in *Arabidopsis* (Orsel *et al.*, 2006; Kotur & Glass, 2015; O'Brien *et al.*, 2016), barley (Tong *et al.*, 2005), rice (Yan *et al.*, 2011), wheat (Taulemesse *et al.*, 2015) and chrysanthemum (Gu *et al.*, 2016). TaNRT2.5 specifically interacted with TaNAR2.1 to transport nitrate in *Xenopus* oocytes, but not with two alternative TaNAR2s. In addition, like AtNRT2.5 (a closely related NRT2; see Fig. S5) TaNRT2.5 does not occur only in root membranes as a 150 kDa molecular complex with AtNAR2.1 *in vivo* (Kotur & Glass, 2015), the TaNRT2.5‐3B protein was unstable when expressed without TaNAR2.1 in the tobacco leaf system (Fig. 3), so TaNAR2.1 may be involved in TaNRT2.5 protein turnover. But unlike *AtNRT2.5* (Lezhneva *et al.*, 2015), overexpressing *TaNRT2.5‐3B* did not increase the 5‐min nitrate influx rate and TaNRT2.5‐3B seemed to locate to the tonoplast in the tobacco leaf system (Fig. 3). This shows that TaNRT2.5‐3B plays a role in nitrate transport to the vacuole and effects intracellular nitrate distribution, therefore having an indirect function in nitrate acquisition from the soil. Increasing the expression of *TaNRT2.1* and *TaNR1* may result in increased 12 h net nitrate uptake rate and nitrate concentration in seedlings of *TaNRT2.5‐3B* overexpression lines (Figs 6a, S10). Expression analysis of the *TaNAR2*s in these overexpressing lines showed that *TaNAR2.1* and *TaNAR2.2* had more transcript, but *TaNAR2.3* did not (Fig. S11). This suggests that, perhaps like *Arabidopsis* AtNRT2.1, the TaNRT2.1 protein may be regulated by the levels of TaNAR2.1 and TaNAR2.2, but not TaNAR2.3. Additionally, overexpressing *TaNRT2.5‐3B* increases post‐anthesis nitrate uptake, resulting in more N accumulated in the aerial parts of the whole plant, including the grain (Fig. 7). In a wheat crop, the late root acquisition of N is known to be important for grain N concentration and quality. Although *TaNRT2.1*, another nitrate transporter, also seems to play a major role in nitrate uptake after anthesis (Taulemesse *et al.*, 2015), the present work suggests that the expression of *TaNRT2.1* in root was slightly enhanced in overexpression lines (Fig. S12a) in high N, and the expression of *TaNRT2.5* in the root increased after anthesis (Fig. 7a). The transcript for nitrate reductase (TaNR1) only increased at high N in the overexpression lines (Fig. S12). These results suggested that *TaNRT2.5* might cooperate with *TaNRT2.1* to play a role in nitrate uptake after anthesis. The present authors’ previous research has already shown that TaNAC2 binds to the promoter of *TaNRT2.1‐6B* and *TaNPF7.1‐6D* and enhances expression of the latter two genes (He *et al.*, 2015). *TaNRT2.1* is root‐specifically expressed and may contribute to nitrate uptake by wheat plants (He *et al.*, 2015). *TaNPF7.1* has been suggested to participate in root nitrate xylem loading and nitrate translocation in shoots (Buchner & Hawkesford, 2014). Taking this information together, *TaNAC2* controls nitrate transport from the soil to grain by regulating *TaNRT2.1*, *TaNPF7.1* and *TaNRT2.5*.

Plants require a large amount of energy during seed germination, so carbohydrate metabolism is increased to power this process (Yu *et al.*, 2014). Alpha‐amylase (α‐d‐1,4‐glucan‐4‐glucanohydrolases) is of critical importance for the breakdown of starch granules during seed germination (Bak‐Jensen *et al.*, 2010; Ju *et al.*, 2019). *TaNRT2.5* transgenic lines have altered *TaAMY* expression and this may have influenced the seed germination rate (Fig. 4). In a previous study, UTP‐glucose pyrophosphorylase (UGPase) was associated with glycogenesis, the synthesis of UDP‐glucose from glucose‐1‐phosphate and UTP, which was upregulated throughout germination (Yu *et al.*, 2014). In the present study, the expression level of *TaUGPase* also was found to increase in *TaNRT2.5‐3B* overexpression lines and decrease in RNAi lines.

**Figure 8:**
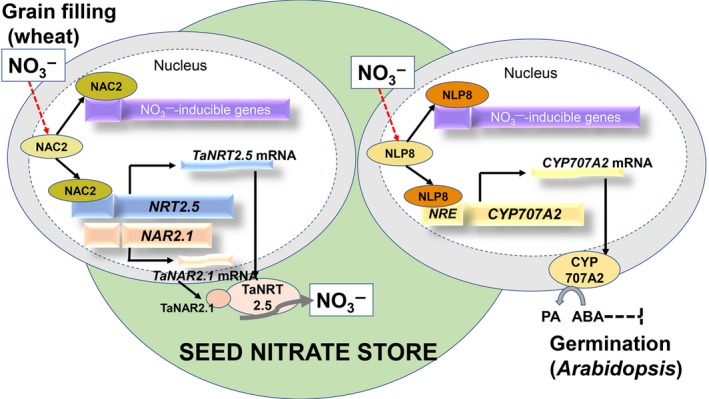
A proposed schematic model for NAC2/TaNRT2.5/TaNAR2.1 activity in nitrate grain filling and subsequent germination integrating data from *Arabidopsis* and wheat. Modified from Yan et al. (2016). Nitrate promotes *TaNAC2* expression to activate the TaNRT2.5/TaNAR2.1 nitrate grain‐filling activity. Nitrate stored in the grain helps provide the signal promoting germination mediated by the abscisic acid (ABA) content controlled by NIN‐like protein 8 (NLP8) and *CYP707A*.

Overexpressing *TaNRT2.5‐3B* increases grain nitrate concentration and germination, suggesting that the transporter may be a functional orthologue of *AtNRT2.7* in *Arabidopsis* (Chopin *et al.*, 2007). The two transporters both show seed‐specific expression pattern, tonoplast localization and nitrate transport activity, but the two genes have some divergent properties. For example, AtNRT2.7 does not require a NAR2 partner protein for the nitrate transport function. The NRT2 genes have functional differences in *Arabidopsis* and wheat although they may be performing the same function in storing nitrate in the seed. The key role of the *TaNAC2*‐*NRT2.5* module in the grain nitrate signal linked to germination is now established in wheat. As in *Arabidopsis* (Yan *et al.*, 2016), there is evidence that ABA might be involved as ABRE *cis*‐acting elements involved in ABA responses can be identified in the promoter regions of both *AtNRT2.7* and *TaNRT2.5* (://bioinformatics.psb.ugent.be/webtools/plantcare/html/). It may be important to separate grain N storage and the signaling role of nitrate for the *TaNAC2*‐*NRT2.5* module. In wheat, increasing *TaNRT2.5* expression by almost 30‐fold (Fig. 4d) did not result in similar fold‐increases in grain fill of N or nitrate. For the RNAi lines too, there was not a huge effect on grain storage of nitrate or N (Fig. 4b, c). Additional factors such as post‐translational regulation might be important, but *TaNAC2‐5A* is regulating the expression of other assimilatory genes that may interact and contribute to the balance of N storage forms in seed. Stored nitrate is important during the grain filling for subsequent germination and this has been summarized in a revised model that includes the novel information provided by the wheat research herein (Fig. 8). In conclusion, *TaNRT2.5* has a specific role in seed nitrate accumulation, an important signal for seed vigour and crop establishment, and the *TaNAC2*‐*NRT2.5* module potentially can be genetically engineered to improve germination rate, seed vigour and grain yield in wheat. TaNAC2 is the transcription factor regulating the ‘workhorse’ TaNRT2.5 transporter which drives nitrate accumulation and, therefore, seedling vigour.

## Author contributions

WL and XH designed and performed most of the experiments; YC contributed to the nitrate transporter activity in *Xenopus laevis* oocyte experiments; YJ, CS and JY performed experiments and interpreted the results; WT, XZ and WH provided technical assistance; MH and HL contributed to the field experiments; AJM and YT supervised the project and designed the experiments; and WL, XH, AJM and YT wrote the manuscript. WL and XH contributed equally to this work.

## Supporting information


**Fig. S1** Seed vigour after artificial aging treatment and seed germination in soil.
**Fig. S2** Seed germination phenotypes of six Chinese wheat varieties.
**Fig. S3** Expression levels of genes in different organs of wheat.
**Fig. S4** Binding abilities of TaNAC2 to the promoter fragments of *TaLAX1‐3B/1D, TaARR12‐6A *and *TaCLCc‐3A*.
**Fig. S5** Phylogenetic tree of NRT2 gene family in *Arabidopsis*, rice, maize, barley, soybean and wheat.
**Fig. S6** Nitrate concentration in the dissected parts of wheat seed.
**Fig. S7** Expression analysis and nitrate transport activity of TaNRT2.5s and TaNAR2s.
**Fig. S8** Gene expression patterns for the *TaNAC2‐5A/B/D* and *TaNRT2.5‐3A/B/D *homeolog genes.
**Fig. S9** *TaNRT2.5* expression influences seed vigour.
**Fig. S10** Nitrate influx rate of *TaNRT2.5‐3B *overexpression lines and WT at the seedling stage
**Fig. S11** Expression levels of *TaNAR2 *genes at seedling stage in roots of *TaNRT2.5‐3B* overexpression lines and WT.
**Fig. S12** Expression levels of nitrate transporter *TaNRT2.1* and nitrate reductase *TaNR1 *at seedling stage in roots of *TaNRT2.5‐3B *overexpression lines and WT.

**Table S1** Primers used in constructs.
**Table S2** Primers used for qRT‐PCR.
**Table S3** Gene information in the ChIP‐seq analysis of TaNAC2.
Please note: Wiley Blackwell are not responsible for the content or functionality of any Supporting Information supplied by the authors. Any queries (other than missing material) should be directed to the *New Phytologist* Central Office.
