# Supplementary material for: A wheat transcription factor positively sets seed vigour by regulating the grain nitrate signal
Source: New Phytol. 2019 Nov 13;225(4):1667–80. doi: 10.1111/nph.16234 (PMC7004088; doi:10.1111/nph.16234)
Supplement: Supplementary file 1 — Fig. S1 Seed vigour after artificial aging treatment and seed germination in soil. Fig. S2 Seed germination phenotypes of six Chinese wheat varieties. Fig. S3 Expression levels of genes in different organs of wheat. Fig. S4 Binding abilities of TaNAC2 to the promoter fragments of TaLAX1‐3B/1D, TaARR12‐6A and TaCLCc‐3A. Fig. S5 Phylogenetic tree of NRT2 gene family in Arabidopsis, rice, maize, barley, soybean and wheat. Fig. S6 Nitrate concentration in the dissected parts of wheat seed. Fig. S7 Expression analysis and nitrate transport activity of TaNRT2.5s and TaNAR2s. Fig. S8 Gene expression patterns for the TaNAC2‐5A/B/D and TaNRT2.5‐3A/B/D homeolog genes. Fig. S9 TaNRT2.5 expression influences seed vigour. Fig. S10 Nitrate influx rate of TaNRT2.5‐3B overexpression lines and WT at the seedling stage Fig. S11 Expression levels of TaNAR2 genes at seedling stage in roots of TaNRT2.5‐3B overexpression lines and WT. Fig. S12 Expression levels of nitrate transporter TaNRT2.1 and nitrate reductase TaNR1 at seedling stage in roots of TaNRT2.5‐3B overexpression lines and WT. [file NPH-225-1667-s001.pdf]

New Phytologist Supporting Information Figs S1–S12

Article title: A wheat transcription factor positively sets seed vigour by regulating the grain nitrate signal

Authors: Wenjing Li, Xue He, Yi Chen, Yanfu Jing, Chuncai Shen, Junbo Yang, Wan Teng, Xueqiang Zhao, Weijuan Hu, Mengyun Hu, Hui Li, Anthony J. Miller and Yiping Tong

Article acceptance date: 22 September 2019

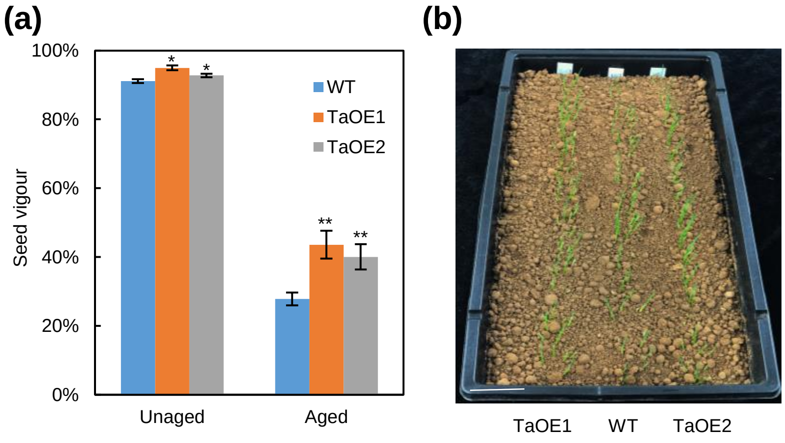

Fig. S1 Seed vigour after artificial aging treatment and seed germination in soil. (a) The seed vigor of wild type and *TaNAC2-5A* overexpression lines were measured after CDT and Tetrazolium assay. Data are means ± SE of three repetitions with 30 seeds each. Significant differences between wild type and transgenic line were according to Student's *t*-test. * P < 0.05, ** P < 0.01. (b) The seed germination in soil pot experiment was assayed using a calcareous soil that was collected from the experimental station of the Institute of Genetics and Developmental Biology in Beijing. The soil was wetted to 20% soil water content, and 100 seeds of the wild type and the TaNAC2-5A transgenic lines were sowed in each pot at 20℃. Photograph was taken, and the shoot length of seedlings was measured at 7 days after sowing. Scale bar = 5 cm.

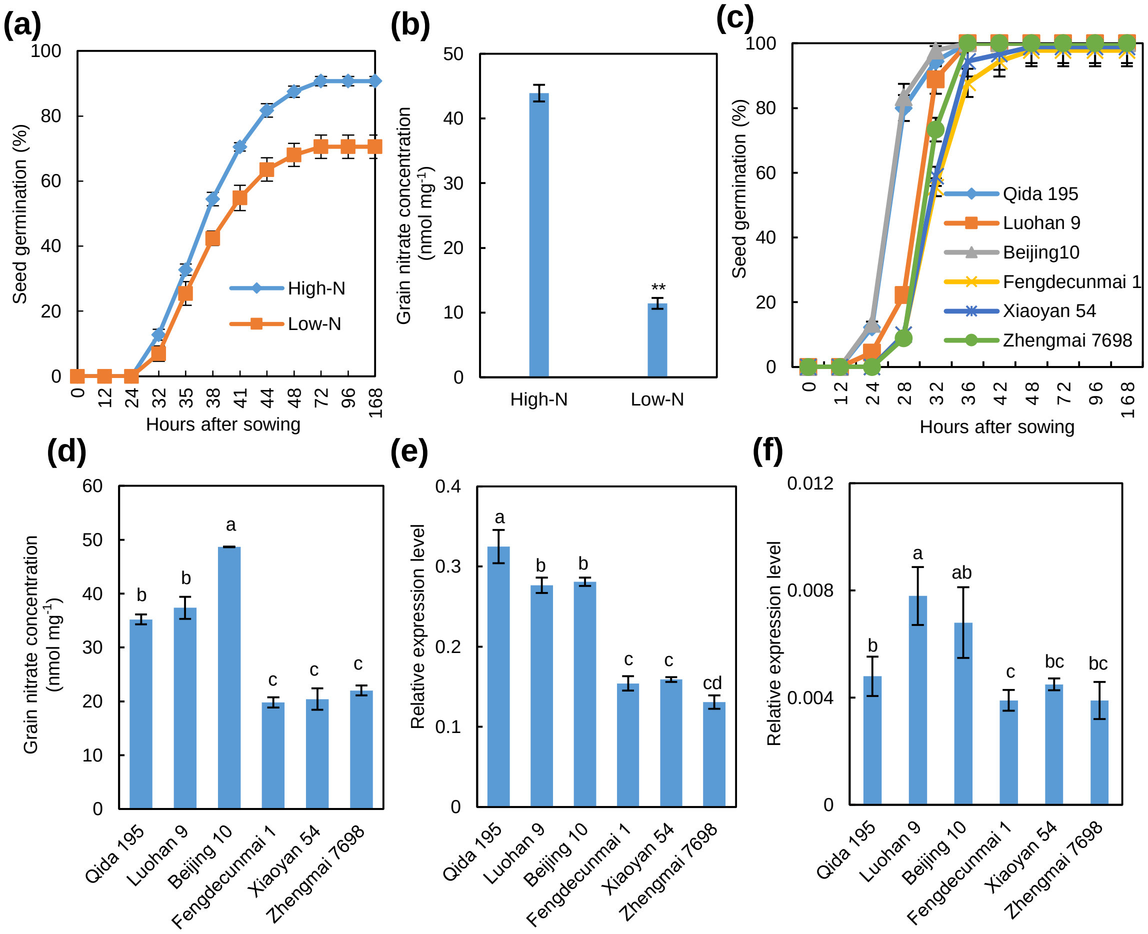

Fig. S2 Seed germination phenotypes of six Chinese wheat varieties. (a and b) Germination time course (a) and grain nitrate concentration (b) in LC23. The seeds were harvested from the plants grown under low N and high N conditions. Germination on Petri dish and grain nitrate measurement were performed one month after harvest. Data represented as means ± SE (n = 4). ** *P* < 0.01 (*t*-test). (c-e) Germination time course (c), grain nitrate concentration (d) and relative *TaNAC2* (e) and *TaNRT2.5* (f) expression in germinating seeds in different wheat verities (Qida 195, Luohan 9, Beijing10, Fengdecun 1, Xiaoyan 54 and Zhengmai 7698). The seeds were harvested from the plants grown high N conditions. One month after harvest, the seeds were used to perform the assays in (c-f). Relative expression level of *TaNAC2* and *TaNRT2.5* was measured in germinating seeds 48 h after sowing. *TaActin* was used as an internal reference. Data represented as means ± SE (n = 4). Different letters in d and e indicate significant difference at *P* < 0.05 (one-way ANOVA).

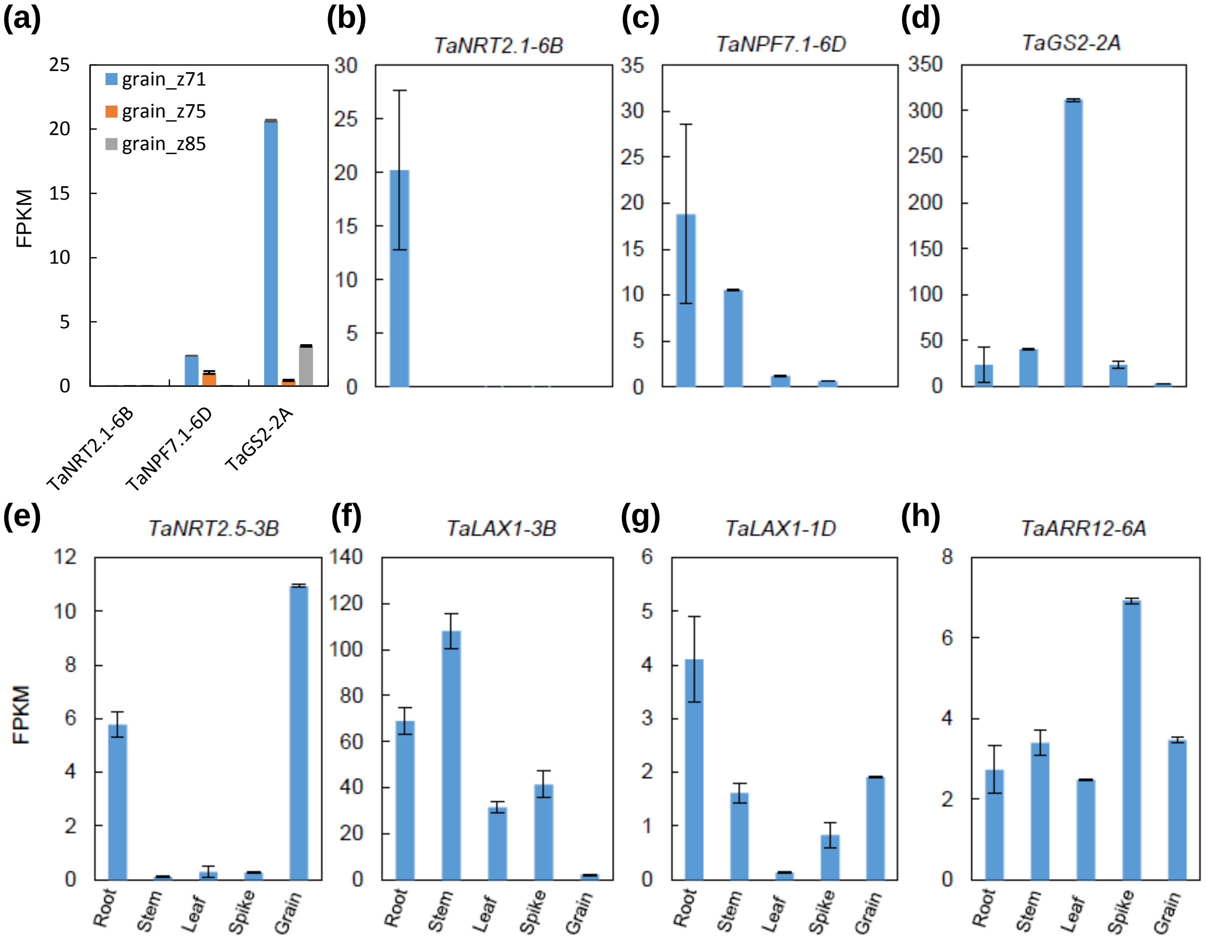

Fig. S3 Expression levels of genes in different organs of wheat. (a) Expression levels of *TaNRT2.1-6B*, *TaNPF7.1-6D* and *TaGS2-2A* in the developing seeds during the grain filling stage. (b-h) *TaNRT2.1-6B* (b), *TaNPF7.1-6D* (c),*TaGS2-2A* (d), *TaNRT2.5-3B* (e), *TaLAX1-3B* (f), *TaLAX1-1D* (g) and *TaARR12-6A* (h) in different organs of wheat. Data were from wheat expression database (https://wheat.pw.usda.gov/WheatExp/). Data are means ± SE (n > 10). FPKM, Fragments per Kilobase of Exon per Million Fragments Mapped

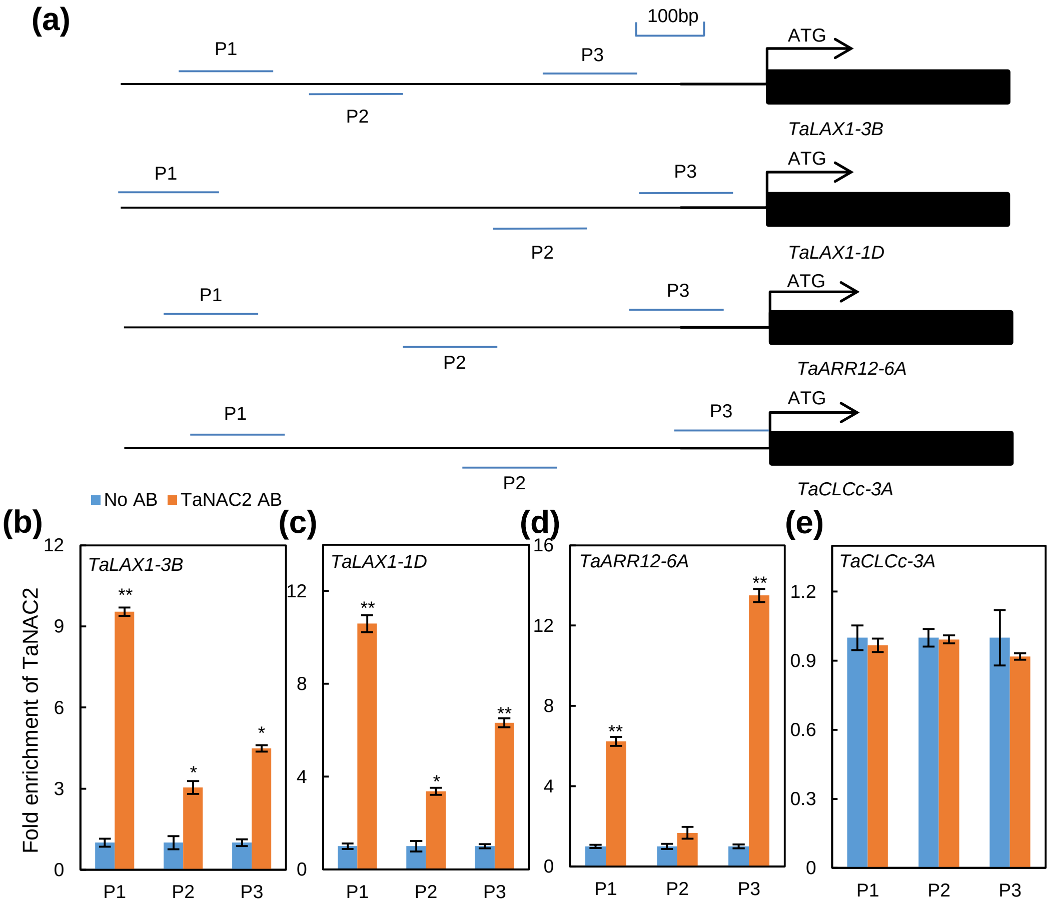

Fig. S4 Binding abilities of TaNAC2 to the promoter fragments of *TaLAX1-3B/1D, TaARR12-6A* and *TaCLCc-3A*. (a) Putative NAC recognition motifs in the promoters of *TaLAX1-3B/1D and TaARR12-6A*. (b-e) Enrichment of the promoter regions in *TaLAX1-3B* (b), *TaLAX1-1D* (c), *TaARR12-6A* (d) and *TaCLCc-3A* (e) recovered in ChIP assays performed with the TaNAC2 antibody using chromatin prepared from WT. Data represented as means ± SE of three replicates. Asterisks indicate that the difference between the means of the TaNAC2-DNA complexes with and without antibody was significant at the $P < 0.05$ (*) and $P < 0.01$ (**) levels by Student *t*-test.

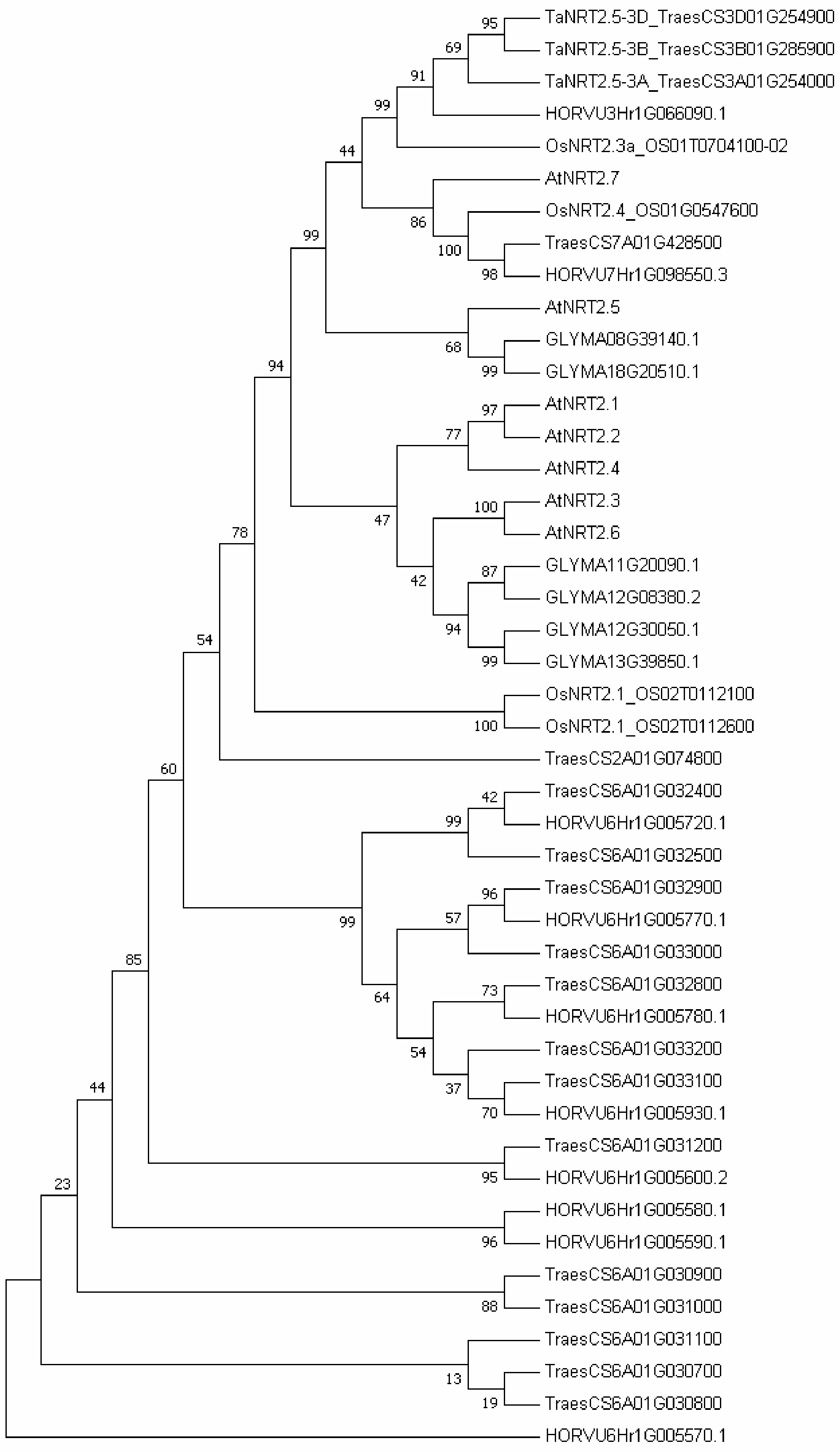

Fig. S5 Phylogenetic tree of NRT2 gene family in *Arabidopsis*, rice, maize, barley, soybean and wheat. The sequences used for phylogenetic analysis include all of the identified TaNRT2 proteins of A genome and three TaNRT2.5s in bread wheat and the NRT2 proteins in *Arabidopsis*, rice, maize, barley and soybean. The phylogenetic tree was constructed using Maximum Likelihood analysis method with Mega 7.0 software.

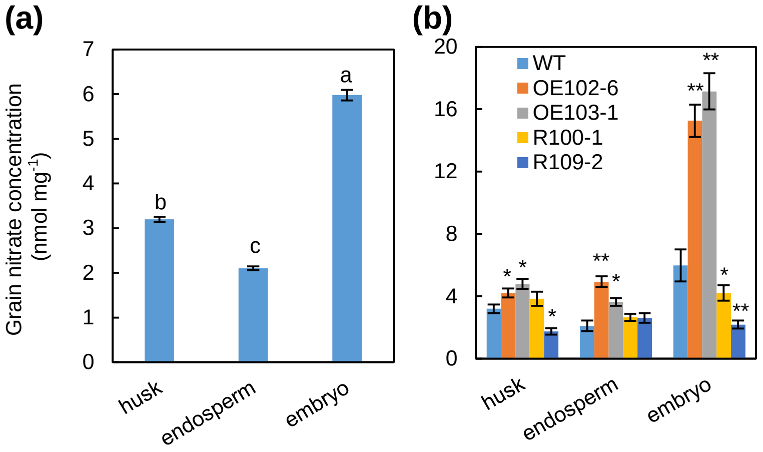

Fig. S6 Nitrate concentration in the dissected parts of wheat seed. (a) Nitrate concentration in the dissected parts in wild type at 28 DPA. Values are means ± SE (n = 4). Different letters indicate significant difference at $P < 0.05$ level according to one-way ANOVA. (b) Nitrate concentration in the dissected parts in *TaNRT2.5* transgenic lines and wild type at 28 DPA. Asterisks indicate that the difference between the means of the *TaNRT2.5* transgenic lines and wild type was significant at the $P < 0.05$ (*) and $P < 0.01$ (**) levels by Student *t*-test.

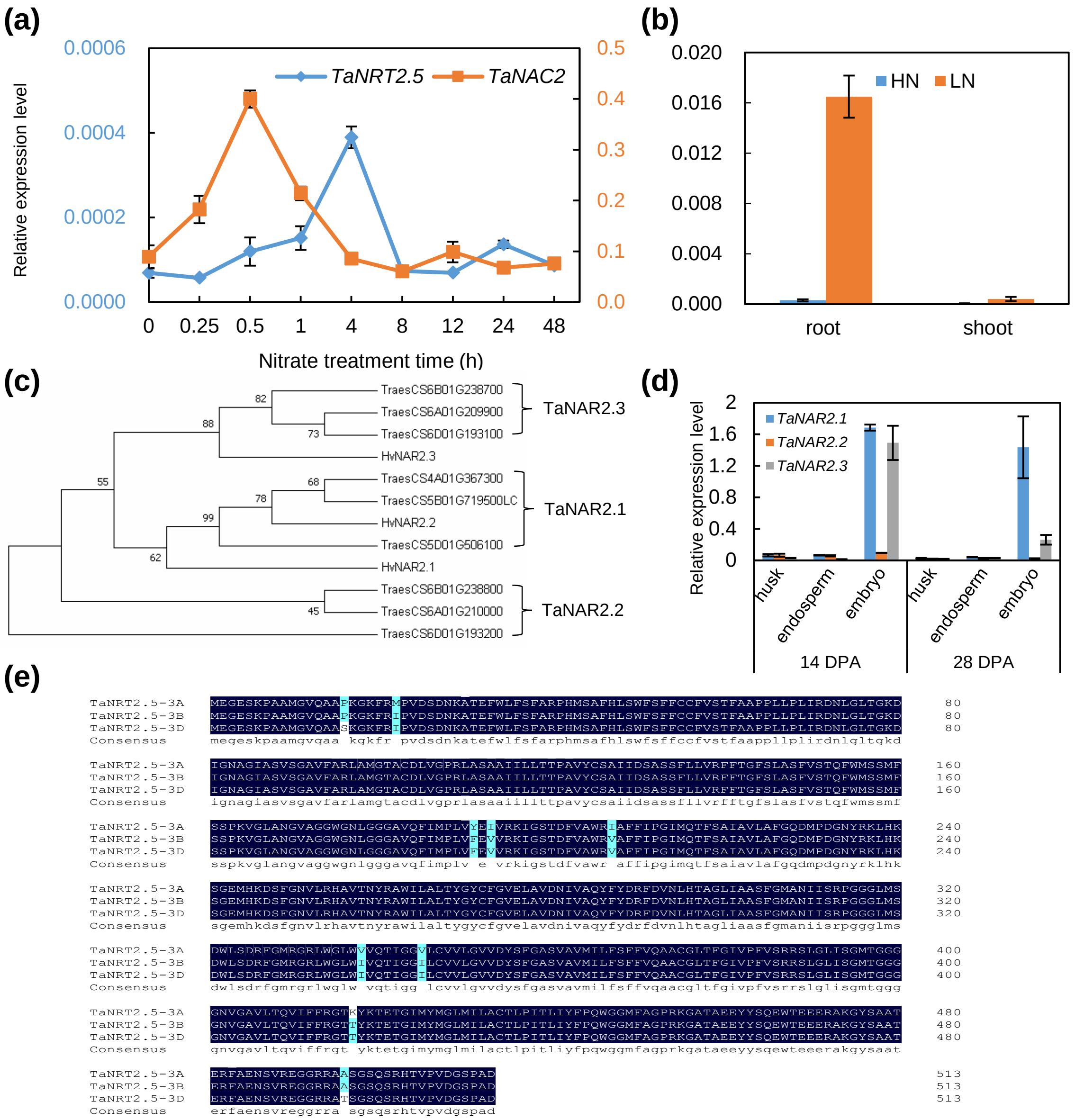

Fig. S7 Expression analysis and nitrate transport activity of TaNRT2.5s and TaNAR2s. (a) *TaNRT2.5* and *TaNAC2* expression in response to nitrate in roots. Data are means ± SE (n = 4). (b) The expression levels of *TaNRT2.5* in shoots and roots of wheat seedlings grown in high N (HN) and low N (LN). Data are means ± SE (n = 4). (c) Phylogenetic tree of NAR2 gene family in barley and wheat using Maximum Likelihood analysis method with Mega 7.0 software. (d) The expression pattern of TaNARs in wheat seeds of 14 and 28 DPA. Data are means ± SE (n = 4). (e) Protein sequence alignment of three TaNRT2.5s in wheat.

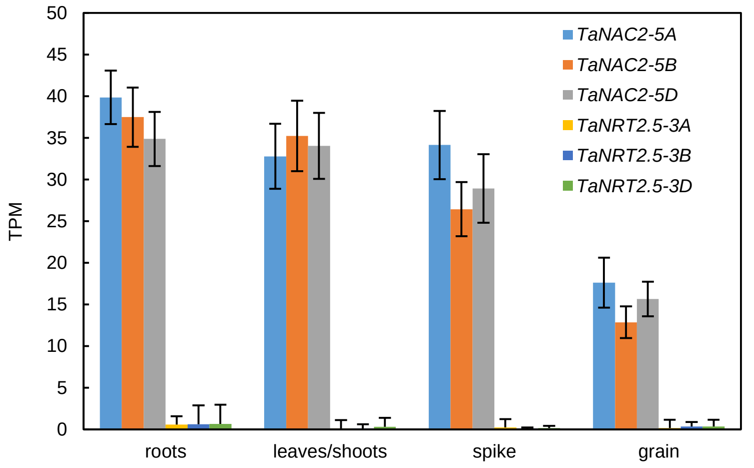

Fig. S8 Gene expression patterns for the *TaNAC2-5A/B/D* and *TaNRT2.5-3A/B/D* homeolog genes. The *TaNAC2-5A/B/D* and *TaNRT2.5-3A/B/D* homeolog genes have the similar expression pattern in different wheat tissues according to the wheat expression database (http://www.wheat-expression.com). Data are means ± SE (n > 10).

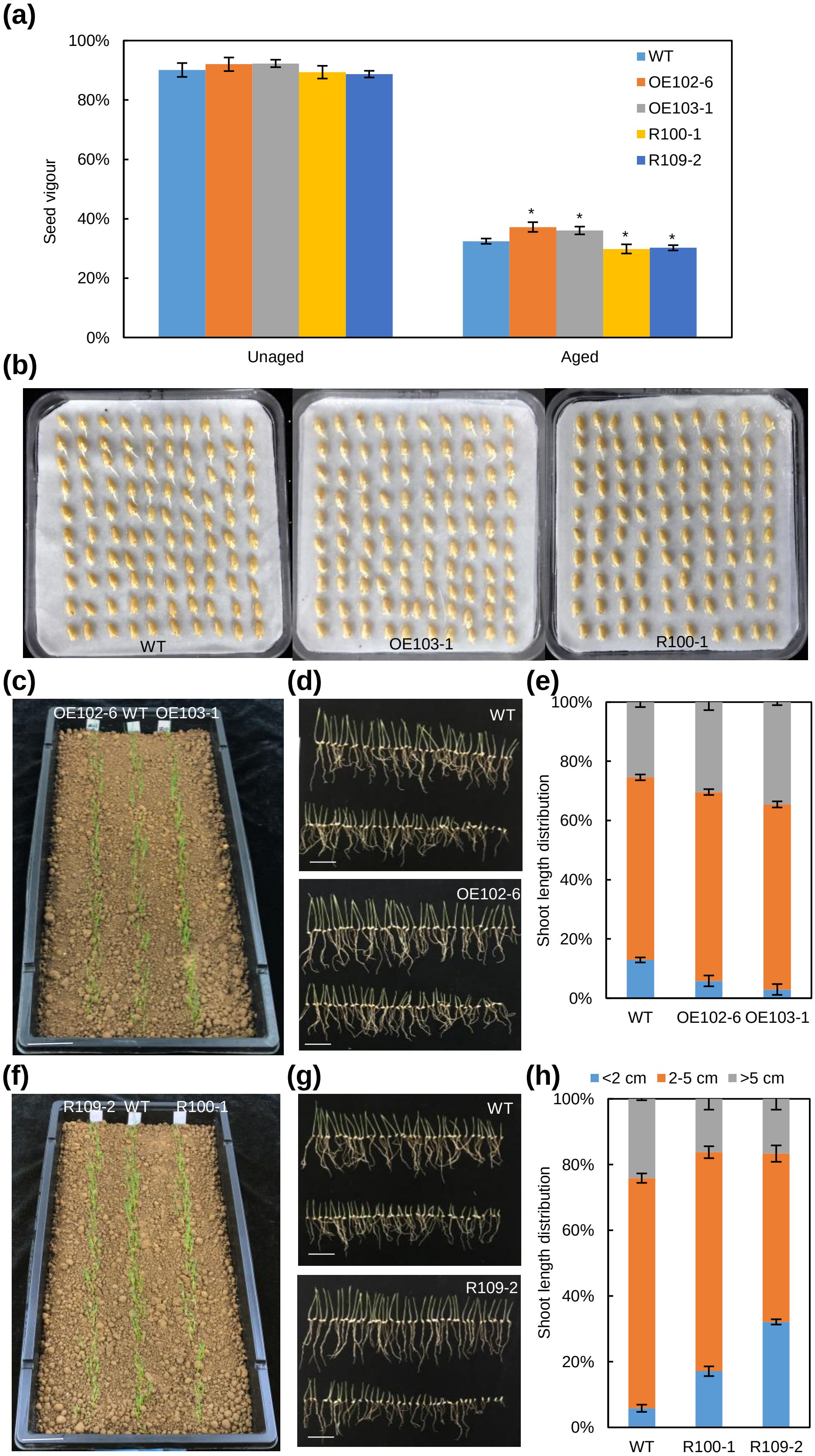

Fig. S9 *TaNRT2.5* expression influences seed vigour. (a) The seed vigour of wild type and *TaNRT2.5* transgenic lines were measured after CDT and Tetrazolium assay. Data are means ± SE of three repetitions with 30 seeds each. Significant differences between wild type and transgenic line were according to Student's t-test. * $P < 0.05$. (b) Photographs of seed germination of the *TaNRT2.5-3B* overexpression and *TaNRT2.5* RNAi lines and WT at 44 h after sowing on Petri dishes. (c-e) Photographs (c and d) and shoot length (cm) distribution (e) of *TaNRT2.5-3B* overexpression lines and WT at 7 days after sowing in soil. Data represented as means ± SE of three replicates. Scales = 5 cm. (f-h) Photographs (f and g) and shoot length (cm) distribution (h) of *TaNRT2.5* RNAi lines and WT at 7 days after sowing in soil. Data represented as means ± SE of three replicates. Scales bar = 5 cm.

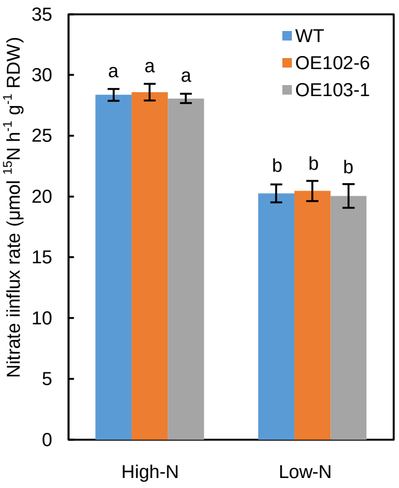

Fig. S10 Nitrate influx rate of *TaNRT2.5-3B* overexpression lines and WT at the seedling stage**.** Nitrate influx rate was measured in roots treated with 2 mM $^{15}NO_3^-$ and 0.2 mM $^{15}NO_3^-$ for 5 min. RDW: root dry weight. Data represented as means ± SE of three replicates. Different letters above the columns indicate significant difference at $P < 0.05$ level (one-way ANOVA).

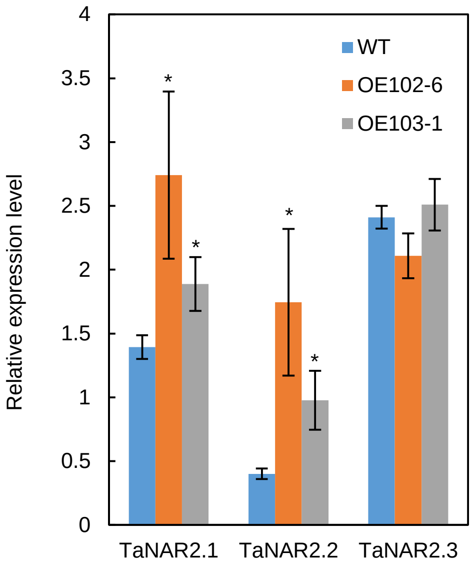

Fig. S11 Expression levels of *TaNAR2* genes at seedling stage in roots of *TaNRT2.5-3B* overexpression lines and wild type. *TaActin* was used as an internal reference. Data were means ± SE of three replicates. Significant differences between wild type and transgenic line were according to Student's *t*-test. * $P < 0.05$.

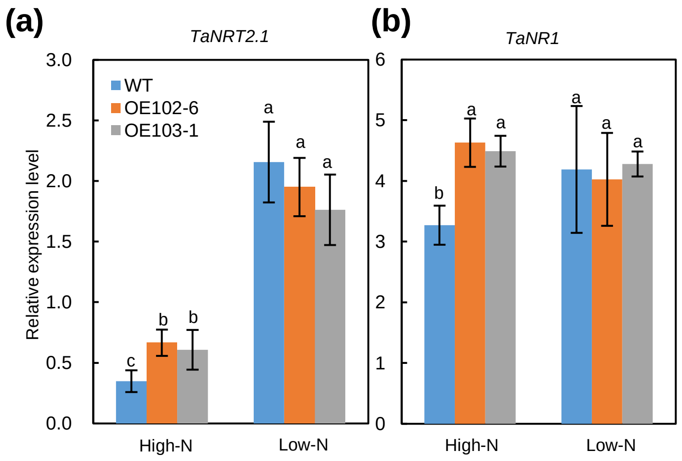

Fig. S12 Expression levels of nitrate transporter *TaNRT2.1* and nitrate reductase *TaNR1* at seedling stage in roots of *TaNRT2.5-3B* overexpression lines and wild type. (a and b) Expression levels of nitrate transporter *TaNRT2.1* (a) and nitrate reductase *TaNR1* (b) in roots at seedling stage of *TaNRT2.5* overexpression lines (OE102-6 and OE103-1) and wild type (WT) grown in a hydroponic culture under high- and low- N conditions. *TaActin* was used as an internal reference. Data were means ± SE of three replicates. Different letters above the columns indicate significant difference at $P < 0.05$ level (one-way ANOVA).
